# Lateral Malleolar Fracture with Concurrent Achilles Tendon Rupture: A Case Report and Literature Review

**DOI:** 10.1155/2020/6479140

**Published:** 2020-02-11

**Authors:** Leslie H. Pyle, Raed W. Al-Gharib, Erik C. Kissel

**Affiliations:** ^1^Podiatric Medicine and Surgery Resident, Detroit Medical Center, 4201 St. Antoine, UHC 9C, Detroit, MI 48201, USA; ^2^Section of Podiatric Surgery, Detroit Medical Center, 6071 W. Outer Drive, Detroit, MI 48235, USA

## Abstract

Achilles tendon and malleolar fractures are commonly seen in isolation, but only a few cases of combined injuries have been reported. In this case, we present a 53-year-old male who sustained an isolated lateral malleolus fracture with an Achilles tendon rupture. Emergency Medicine physicians should consider the possibility of these injuries in combination.

## 1. Introduction

It is estimated that 3.7 million people with exercise-induced or sports-related injuries present to the Emergency Department each year [1]. Approximately, 60% of these injuries are thought to involve the lower limb, with 22–50% resulting from isolated ankle trauma [1]. Emergency Medicine physicians are typically first in line to evaluate and diagnose acute lower extremity injuries. However, it has been shown that fractures, soft tissue injuries, and subluxations are some of the most frequently missed pathologies in the Emergency Department [2].

Ankle fractures can be detrimental to an individual, as damage typically occurs to both osseous and ligamentous structures. Only a small deviation in the tibiotalar joint can result in pain and lifelong arthritis [3]. Identification of these injuries is critical. Several classification systems have been developed to assist in identification of these injuries—Danis–Weber, AO-Muller-Orthopaedic Trauma Association (AO/OTA), and Lauge-Hansen.

The Danis–Weber classification was first described by Robert Danis in 1949. It was modified and popularized by Bernhard Georg Weber in 1972 [4]. This classification was developed as a method for describing ankle fractures that focus primarily on the fibula, by correlating the proximity of the fibular fracture relative to the distal ankle syndesmosis. The AO/OTA classification was developed as an expansion to the Danis–Weber classification, to include the location of the fracture and degree of comminution [4]. The Lauge-Hansen classification was developed by utilizing a cadaveric specimen by a Danish surgeon, Niel Lauge-Hansen [4]. It utilizes the position of the foot at the time of injury and the direction of the deforming force. Based on these classification systems, a supination-external rotation injury, synonymous with a Weber B fracture, accounts for approximately 40–70% of all ankle fractures [5].

Soft tissue injuries such as Achilles tendon ruptures can also lead to debilitating outcomes when left untreated. This injury is most often seen in middle-aged individuals participating in athletic events. Three common mechanisms leading to Achilles tendon rupture include unexpected ankle dorsiflexion, violent dorsiflexion of a plantarflexed ankle, or pushing off a weightbearing foot with knee extended [6]. Approximately, 25% of acute Achilles tendon ruptures are missed at initial presentation [7].

When considering foot and ankle trauma, Achilles tendon injuries and ankle fractures are common in isolation. It has been postulated by multiple authors that a malleolar fracture with an Achilles tendon rupture is infrequently seen. These injuries were first perceived in association with alpine skiing [8–10]. To date, only thirteen cases of a malleolar fracture occurring simultaneously with an Achilles tendon rupture have been reported [11–23]. The majority of these injuries involved an isolated medial malleolar fracture [11–18, 20, 20, 21, 23].

In this report, we present the even rarer occurrence of an isolated lateral malleolar fracture with an Achilles tendon rupture, with only one previous case identified in the literature [19]. Emergency Medicine physicians should consider the possibility of these injuries in combination.

## 2. Case Report

**Figure 1:**
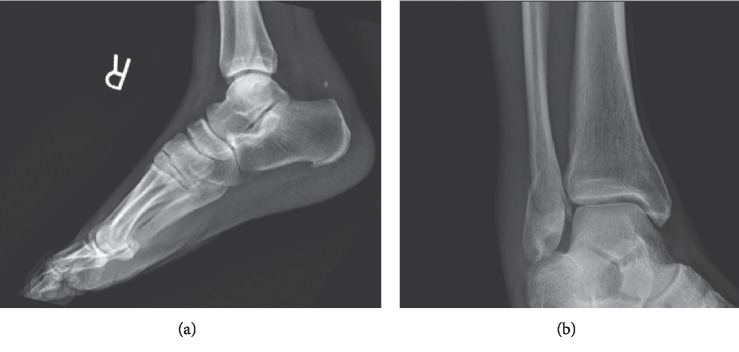
Plain radiographs: (a) lateral view and (b) mortise view.

**Figure 2:**
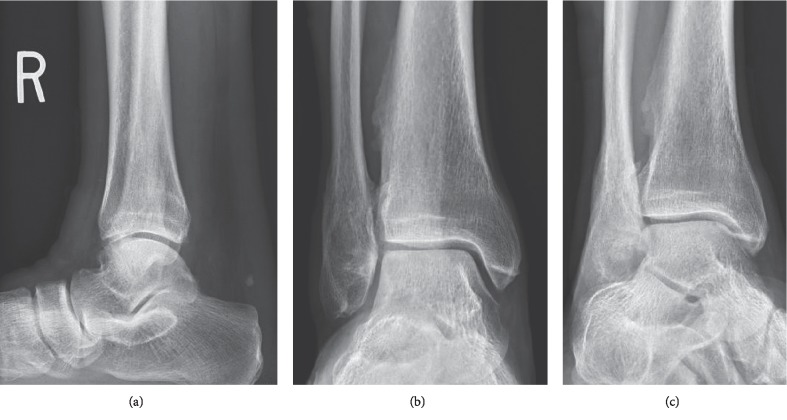
Plain radiographs: (a) lateral view, (b) anteroposterior view, and (c) mortise view.

A 53-year-old male presented to the Emergency Department after slipping on ice and falling down three concrete steps. He did not recall the specific mechanism of injury due to intoxication. On physical exam, significant nonpitting edema was observed to the lateral and posterior aspects of the ankle. There was pain with palpation to the lateral malleolus. A dell was palpated 4 cm proximal to the Achilles tendon insertion. Anteroposterior, mortise, and lateral radiographs were obtained which demonstrated a Weber B fracture of the lateral malleolus and obliteration of the pre-Achilles fat pad (Kager's triangle), suspicious for tendon injury (Figure 1). The patient was placed in a plantarflexed posterior splint in the Emergency Department. He was given crutches and made nonweightbearing, but he was lost to follow-up. One year later, the patient presented to the clinic for evaluation. On physical exam, a nonpainful, palpable bulbous thickening was appreciated at the prior Achilles tendon injury site. No functional deficits from either injury were appreciated as posterior muscle compartment strength and ankle joint range of motion were noted to be equal and symmetric bilaterally. Anteroposterior, mortise, and lateral radiographs were obtained which demonstrated a healed, malunited Weber B fracture of the lateral malleolus. The pre-Achilles fat pad was normal in appearance (Figure 2).

## 3. Discussion

An extensive literature search using the MEDLINE and Google Scholar databases resulted in thirteen case reports detailing an ankle fracture combined with an Achilles tendon rupture (Table 1). Twelve of these cases involved a fracture of the medial malleolus. The proposed mechanism of injury for the Achilles tendon rupture is largely agreed upon by these authors, suggesting this injury resulted from a sudden upward force applied to the forefoot, such as with hyperdorsiflexion. The mechanism causing the medial ankle fracture, on the other hand, has several cited mechanisms—hyperextension of the ankle [11, 15], hindfoot eversion [12, 14], or hindfoot inversion [11, 14–16]. Despite these differences, the Achilles tendon rupture very likely occurred first, followed by the medial malleolus fracture.

In regard to our present case, there is only one other reported in the current literature which was reported to have resulted while jumping continuously on the same leg during exercise [19]. Dinato et al. suggested that the resulting injury occurred through combined eccentric and concentric forces that were sustained during exercise that consisted of rapid acceleration and deceleration with the knee extending, resulting in the observed fracture and concurrent rupture of Achilles tendon [19]. As our reported case occurred from a fall down concrete steps, we believe our injury resulted similarly to the previously reported mechanisms. A forced hyperdorsiflexion of the foot in a middle-aged individual likely resulted in the Achilles tendon rupture, followed by axial loading of the inverted hindfoot, leading to the isolated fibular fracture.

Moonen et al. attribute diagnostic errors in the Emergency Department with insufficient history-taking and inadequate physical exams [2]. In six of the thirteen cases, either the ankle fracture or Achilles tendon rupture was initially missed [12, 13, 17, 18, 20, 21]. Four of the six cases missed an Achilles tendon rupture [12–14, 18]. The Achilles rupture was found as early as several days [18] and as late as 5 weeks after injury [12]. The remaining two cases missed a medial malleolus fracture, which were both discovered on postoperative X-ray after an Achilles tendon repair [20, 21].

This case report provides insight into an atypical injury. Achilles tendon ruptures in combination with ankle fractures are rarely observed. A review of the literature reveals that when these injuries present concurrently, one of the two injuries is frequently missed on initial presentation. Missed or delayed diagnosis can have lasting effects on patients, and Emergency Medicine physicians should consider the possibility of these injuries in combination.

## Figures and Tables

**Table 1:** Previous case reports in English language literature.

Author	Year	Age (years)	Gender	Cause of injury	Type of fracture
Martin and Thompson [18]	1986	61	M	MVA	Medial malleolus
Barron and Yocum [12]	1993	30	F	Gymnastics	Medial malleolus
Pieper et al. [21]	1998	45	M	Basketball	Medial malleolus
Lubin et al. [16]	2000	40	M	Fall from height	Medial malleolus
Assal et al. [11]	2002	35	M	Fall from ladder	Medial malleolus
Garneti et al. [14]	2005	42	M	Fall down stairs	Medial malleolus
Maffulli and Richards [17]	2006	38	M	Fall from tree	Medial malleolus
Tanaka and Shimizu [22]	2008	32	M	Jump from truck	Medial malleolus
Dinato et al. [19]	2010	39	F	Exercise injury	**Lateral malleolus**
Turkmensoy et al. [23]	2013	42	M	Fall down stairs	Medial malleolus
Lu and Maruo Holledge [15]	2016	59	M	Fall from step	Medial malleolus
Nakajima et al. [20]	2016	45	M	Futsal	Medial malleolus
Elmajee et al. [13]	2017	30	F	Rock climbing	Medial malleolus
Present report	2020	54	M	Fall down concrete steps	**Lateral malleolus**
